# The gene encoding the insulin-like androgenic gland hormone in an all-female parthenogenetic crayfish

**DOI:** 10.1371/journal.pone.0189982

**Published:** 2017-12-20

**Authors:** Tom Levy, Ohad Rosen, Ohad Simons, Amit Savaya Alkalay, Amir Sagi

**Affiliations:** 1 Department of Life Sciences, Ben-Gurion University of the Negev, Beer-Sheva, Israel; 2 The National Institute for Biotechnology in the Negev, Ben-Gurion University of the Negev, Beer-Sheva, Israel; Shanghai Ocean University, CHINA

## Abstract

Male sexual differentiation in crustaceans is controlled by the androgenic gland (AG), a unique male endocrine organ that, in decapods, is located at the base of the 5th pereiopod. In these animals, the insulin-like androgenic gland hormone (IAG) is the major factor secreted from the AG to induce masculinization and maintain male characteristics. It has, however, recently been proposed that this hormone also plays a role in growth and ovarian development in females. In this study, we tested such a possibility by searching for the *IAG* gene in the marbled crayfish, a parthenogenetic animal that reproduces asexually to form an all-female genetic clone. Based on the phylogenetic relationship between the marbled crayfish and *Procambarus fallax*, a gonochoristic species of the same North American Cambaridae family, we searched for the *IAG* gene in the marbled crayfish and then fully sequenced it. The open reading frame of the gene was found to be completely identical in the two species, and their introns shared over 94% identity. It was also found that, in addition to its expression at the base of the 5th pereiopod and in the testes of male *P*. *fallax* crayfish, IAG was expressed in the muscle tissue of *P*. *fallax* males and females and even of the parthenogenetic marbled crayfish. These findings provide new insight into possible functions of IAG, in addition to its role as a masculinization-inducing factor, and also constitute the basis for a discussion of the evolutionary relationship between the above two species.

## Introduction

In crustaceans, male sexual differentiation is fundamentally controlled by the androgenic gland (AG), a unique male crustacean endocrine organ [1, 2]. Since the discovery of the AG by Cronin [3], it has been regarded as the major player in the crustacean masculinization process [1, 4–6]. This notion was supported by the findings that AG implantation in females caused masculinization [7], while andrectomized males exhibited feminization [8, 9]. In keeping with these findings, it was also shown that a single injection of AG cell suspension caused full sex reversal of females into males [10]. In decapod crustaceans, the AG is known to secrete the insulin-like androgenic gland hormone, designated IAG [5]. It is believed that this hormone is a key masculine AG factor, because knocking down its encoding gene–by dsRNA injections–caused full sex reversal of males into females [11]. Although the *IAG* gene has been found in the genome of both genders in gonochoristic species, it was originally believed to be expressed exclusively in the male AG. However, a few recent studies have suggested that, despite its prominent role as a masculinization-inducing hormone, IAG also stimulates growth and ovarian development in females [12, 13].

While IAG has been found in females of gonochoristic species, mining for the *IAG* gene in a parthenogenetic (all-female) crustacean has never been attempted, although such a step would make a significant contribution to characterizing the role of IAG in female crustaceans. A particularly suitable model for such a study is the marbled crayfish (Marmorkrebs; *Procambarus fallax* f. *virginalis*), which is a parthenogenetic crayfish that was discovered in the aquarium trade in the 1990s [14]. The marbled crayfish exhibits a virginal form of reproduction in which a single female produces genetically identical all-female progeny [15]. The marbled crayfish belongs to the North American Cambaridae family [16] and is most closely related to *P*. *fallax*, with the currently held premise being that the marbled crayfish might be the parthenogenetic form of *P*. *fallax* [17]. However, it has recently been reported that, unlike the diploid *P*. *fallax*, the marbled crayfish is a triploid organism and hence an independent species [18].

In this study, we found, for the first time, the AG of *P*. *fallax* and sequenced its *IAG* gene, designated *Pf-IAG*. We then constructed a one-shot genomic library of the marbled crayfish and, in light of the evolutionary relationship between the two species, we searched for the *IAG* gene in the genome of the marbled crayfish. Later, we examined the IAG expression pattern in *P*. *fallax*–both males and females–and in the marbled crayfish. The findings shed light on the role of IAG in female decapods, with important evolutionary implications.

## Materials and methods

### Animals

*P*. *fallax* animals were collected from St. Johns River (Florida, USA) on the basis of their morphological characteristics. To confirm that the collected animals were indeed *P*. *fallax*, molecular genetic analysis was performed using partial sequences as markers of mitochondrial protein coding cytochrome oxidase subunit I (COI) and mitochondrial 12S ribosomal RNA, as previously described [17]. The *P*. *fallax* crayfish were held in aquaria (80 L), while marbled crayfish from the aquarium trade were grown and maintained in 600-L tanks, both at Ben-Gurion University of the Negev, Beer-Sheva, Israel. The aquaria and tanks were supplied with constant aeration, water was recirculated through a biofilter, and the animals were fed *ad libitum*.

### Histology

Sperm ducts (vas deferens) and 5th pereiopods were dissected from the genetically validated *P*. *fallax* males, and tissue samples were fixed as previously described [10]. Samples were gradually dehydrated through a series of increasing alcohol concentrations, incubated with xylene, and embedded in Paraplast (Kendall, Mansfield, MA, USA) according to conventional procedures. Consecutive sections of 5 μm were placed on silane-coated slides (Menzel-Gläser, Braunschweig, Germany) and stained with hematoxylin and eosin for morphological observations as follows: slides were dipped in Xylene for 5 min × 2, then 100%, 90%, 80% and 70% of EtOH for 1 min each. Later, in tap water for 1 min, Hematoxylin for 5 min, tap water for 3 min and Acidic 70% EtOH for 10 sec. The final stage included dipping the slides in Eosin, 95% and 100% EtOH for 5 min × 2 each, Xylene for 5 min × 2 and then cover the section with cover slip.

### Sequencing *Pf-IAG* mRNA and bioinformatics analysis

Total RNA was extracted from the 5th pereiopods of several *P*. *fallax* males using EZ-RNA Isolation kit (BI, Cromwell, CT, USA), and cDNA was prepared using qScript cDNA Synthesis kit (Quanta, Beverly, MA, USA) according to the manufacturer's protocols. A forward degenerative primer (5’-GATCAGRTHGACTTYGACTGYGG-3') was designed on the basis of the conserved amino acids sequence DFDCG in the IAG B-chains of *Cherax quadricarinatus*, *C*. *destructor* and *P*. *clarkii* (accession numbers DQ851163.1, EU718788.1 and KT343750.1, respectively). The *Pf-IAG* mRNA sequence was obtained by the rapid amplification of cDNA ends (RACE) method using SMARTer RACE cDNA Amplification kit (Clontech, Mountain View, CA, USA), with the above-mentioned forward degenerative primer and the Universal Primers Mix (UPM) from the RACE kit (including the long universal primer: 5'-CTAATACGACTCACTATAGGGCAAGCAGTGGTATCAACGCAGAGT-3', and the short universal primer: 5'-CTAATACGACTCACTATAGGGC-3'). Additional PCR amplification of the 5' and 3' regions was performed using specific primers (3RACE_For: 5'-CTGTCCGGGTTCCATCAGTTGTA-3', 5RACE_Rev: 5'- GTCCAAGATGGTCACAGTGGCTG-3') and the UPM with the above mentioned RACE kit. After sequencing the full *Pf-IAG* mRNA, the predicted structure of the protein was inferred from its deduced amino acids sequence. In addition, multiple sequence alignment (MSA) of Pf-IAG with IAG peptides from 17 different crustacean species (S1 Table) was performed using the CLUSTAL W algorithm. Phylogenetic analysis was performed by the Neighbor-Joining method [19] with bootstrapping 1000 replicates using MEGA version 6.0 [20].

### Sequencing the genome of the marbled crayfish

DNA was extracted–using the Qiagen DNeasy Blood & Tissue Kit (Qiagen, Venlo, Netherlands) according to the manufacturer's instructions–from the hemolymph of an adult marbled crayfish. A one-shot genomic library for this animal was constructed by next generation sequencing (NGS) using a MiSeq apparatus (Illumina, San Diego, CA). The raw reads were filtered by removing adapter sequences, contaminations, and low-quality reads. *De-novo* assembly using the CLC genomic workbench 7.3 (CLC-Bio, Aarhus, Denmark) was performed with default parameters to construct the above-mentioned genomic library.

### Sequencing the *IAG* gene of *P*. *fallax* and the marbled crayfish

**Fig 1:**
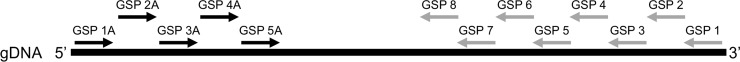
Primers used for genome walking. The schematic order of the primers used for genome walking upstream (gray arrows) and downstream (black arrows) is represented.

Based on the phylogenetic relationship between *P*. *fallax* and the marbled crayfish, we performed a nucleotide BLAST alignment of *Pf-IAG* mRNA against our newly established marbled crayfish genomic library. From the two contig fragments that were found in the library, we amplified and extended the *Pf-IAG* gene by genome walking, as previously described [21], using the Universal GenomeWalker^TM^ 2.0 (Clontech Laboratories Inc, Mountain View, CA) with the conditions mentioned in the manufacturer's protocol and specific primers as listed in Table 1. The order of the primers for genome walking is schematically represented in Fig 1. Sanger sequencing [22] of the amplified fragments was conducted.

**Table 1:** Primer sequences used in this study.

Primer name	Purpose	Primer sequence 5’ → 3’
Degenerative primer	Forward degenrative primer for RACE	GATCAGRTHGACTTYGACTGYGG
Long UPM	Universal RACE primer	CTAATACGACTCACTATAGGGCAAGCAGTGGTATCAACGCAGAGT
Short UPM	Universal RACE primer	CTAATACGACTCACTATAGGGC
3RACE For	Amplification of 3’ cDNA end	CTGTCCGGGTTCCATCAGTTGTA
5RACE Rev	Amplification of 5’ cDNA end	GTCCAAGATGGTCACAGTGGCTG
MK IAG #13 For	Spatial expression of IAG	CCTTCTGGTGGACTTCGACT
MK IAG #1 Rev	Spatial expression of IAG	TTGTCTGTGTTGGCTTGACG
Marmorkrebs 12S For	Housekeeping gene	TGGCGGTGTTTTAGTCTAGTT
Marmorkrebs 12S Rev	Housekeeping gene	ACTTATAAGCTGCACCTTGATCT
MK GSP1	5’ GSP outer primer for genome walking	CCGGGTATTATGGCTTCTGGAGGCTC
MK GSP2	5’ GSP nested primer for genome walking	TCTGTGTCTTGACGAAGGTGTGAGCG
MK GSP3	5’ GSP nested primer for genome walking	GTTATGTACAGAGCAACGTCCTGACG
MK GSP4	5’ GSP nested primer for genome walking	GTCAACAAGAGTGATGTCCTTAGCGGG
MK GSP5	5’ GSP nested primer for genome walking	TCAAGCCTTGTTGATTTAGGACCAGCC
MK GSP6	5’ GSP nested primer for genome walking	CATATACTGTCCATCGTGTCCGCCAG
MK GSP7	5’ GSP nested primer for genome walking	TTCCACCCAGTACGAGGAAGATGGCA
MK GSP8	5’ GSP nested primer for genome walking	GAGCATCGTTGAGTTACAGCAGGAGG
MK GSP1A	3’ GSP outer primer for genome walking	CCCTCTGCACTCCTCCGGCGGCACTC
MK GSP2A	3’ GSP nested primer for genome walking	CCTATCCCAACTGGCGGCCCCCTCAC
MK GSP3A	3’ GSP nested primer for genome walking	GCTCACACCTTCGTCAAGACACAGAC
MK GSP4A	3’ GSP nested primer for genome walking	ACTACAGACAGGGAGCCACAAACACG
MK GSP5A	3’ GSP nested primer for genome walking	AAACAACTATTCACGTCCCACTCCCG

### *IAG* expression in *P*. *fallax* and marbled crayfish tissues

Since the *IAG* gene is known as a growth and reproduction controlling factor [5, 12, 13, 23], somatic and reproductive related tissues were dissected from three *P*. *fallax* males, six marbled crayfish and one *P*. *fallax* female that served as a control. The following tissues were dissected: base of the 5th pereiopods, gonads, hepatopancreas, muscle tissue, and cuticle. Total RNA was extracted from the tissues, followed by synthesis of cDNA as previously described [10]. cDNA, 1 μL, was amplified by PCR (94°C for 3 min, followed by 35 cycles of 94°C for 30 s, 59°C for 30 s, and 72°C for 45 s, and then a final elongation step of 72°C for 10 min) with 1 μL of forward primer, 1 μL of reverse primer, 10 μL of Ready Mix REDTaq (Sigma, St. Louis, MO, USA) and water to a final volume of 20 μL. Spatial expression of *Pf-IAG* (accession number KX619618.1) was performed with the following specific primers: MK_IAG_#13_For: 5'-CCTTCTGGTGGACTTCGACT-3' and MK_IAG_1#_Rev: 5'-TTGTCTGTGTTGGCTTGACG-3'. Expression of 12S rRNA (HM358015.1), (known as an established marker in crayfish [17], served as a positive control using the following specific primers: Marmorkrebs_12S_For: 5'-TGGCGGTGTTTTAGTCTAGTT-3' and Marmorkrebs_12S_Rev: 5'-ACTTATAAGCTGCACCTTGATCT-3'. PCR products were separated on 2% agarose gels, stained with ethidium bromide, and visualized on a UV table.

## Results

### Characterization of the AG in *P*. *fallax*

**Fig 2:**
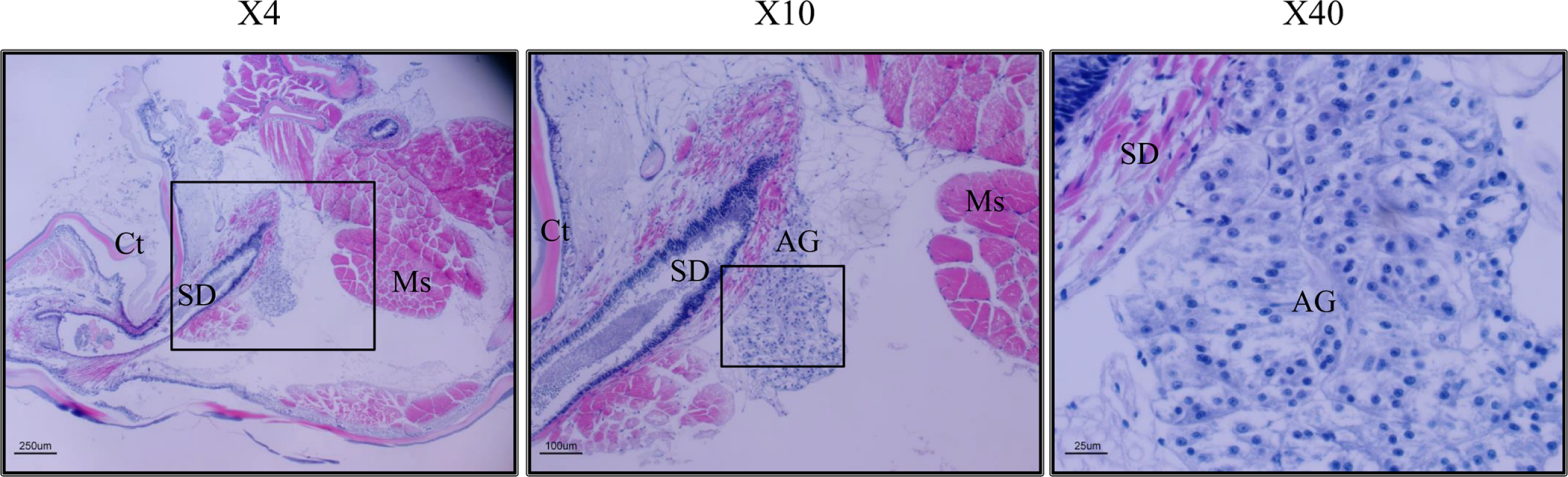
Histological section from the base of the 5th pereiopod of a *P*. *fallax* male. Three different enlargements are presented (×4, ×10 and ×40). The exact area presented in the enlargement in the right panel is surrounded by a black frame in the left and middle panels. Sperm duct (SD), androgenic gland (AG), cuticle (Ct), and muscle (Ms). The sections were stained with H&E.

Histological sections from the base of the 5th pereiopod of *P*. *fallax* (Fig 2) showed the AG as a cord of epithelial cells adjacent to the terminal segment of the sperm duct. The representative section clearly confirmed the location of the AG in *P*. *fallax* similar to other crayfish species [5].

### Characterization of the IAG hormone in *P*. *fallax*

**Fig 3:**
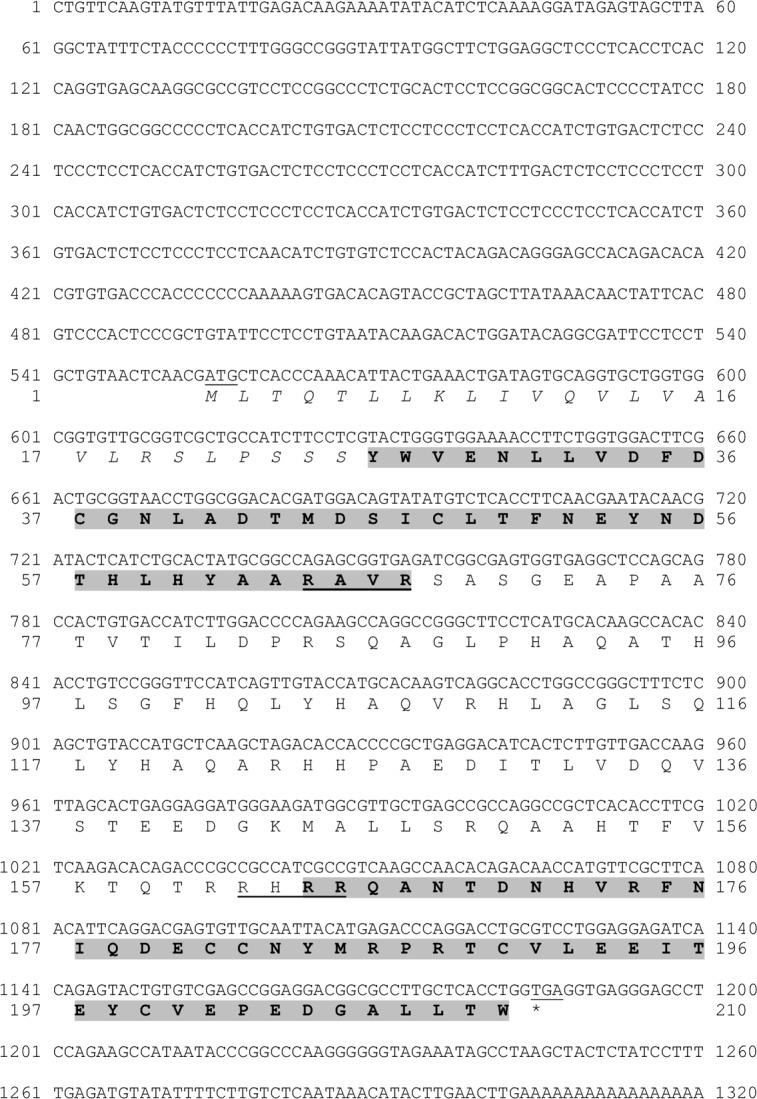
Predicted structure of Pf-IAG. The full sequence of *Pf-IAG* mRNA and its open reading frame (ORF)-deduced amino acids. The signal peptide is shown in italics. B (first) and A (second) chains are marked with a gray background, with C peptide, including its cleavage sites (underlined), flanked between them. The start codon (ATG) is underlined, and the stop codon (TGA) is underlined and indicated with an asterisk.

**Fig 4:**
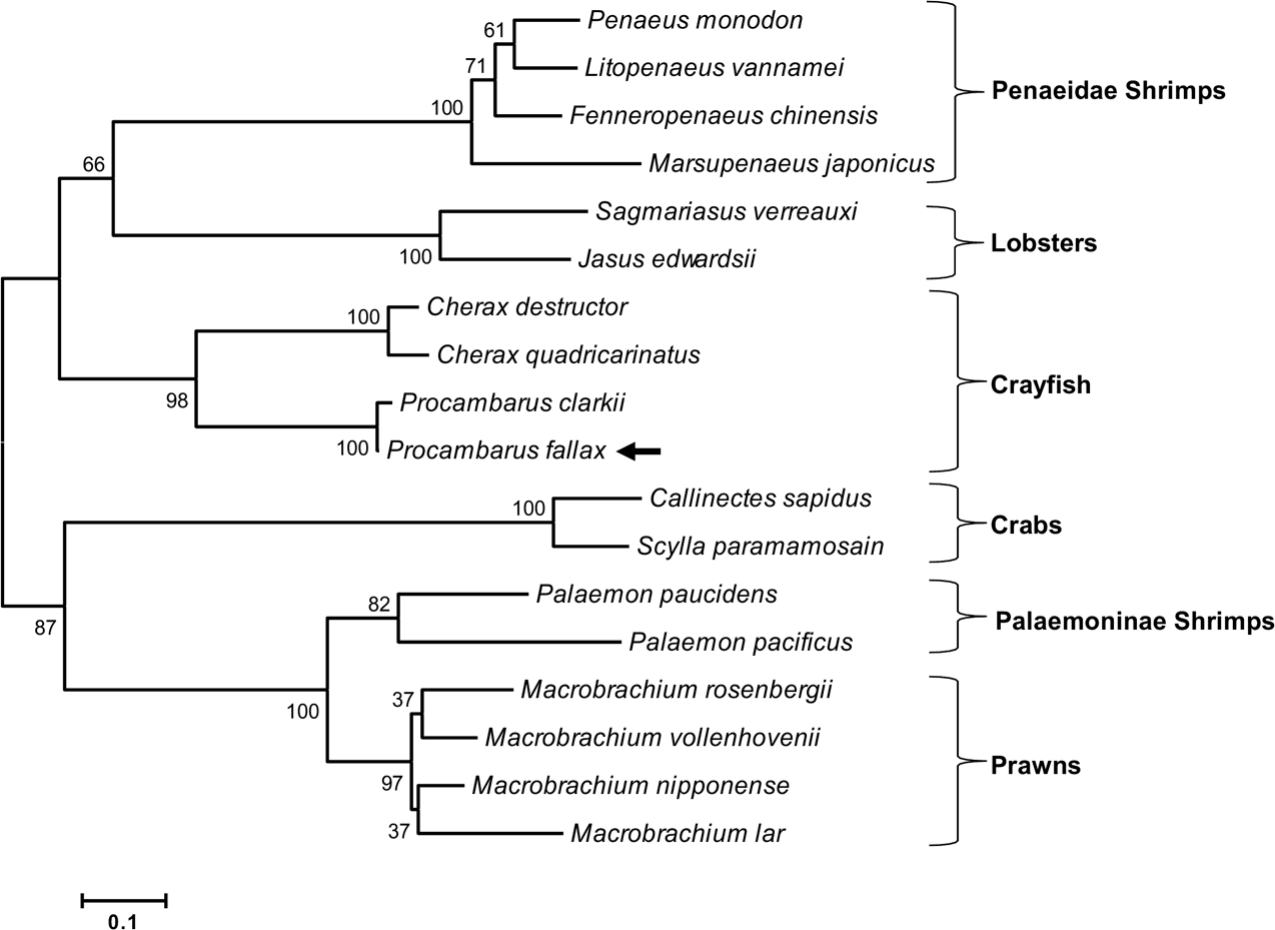
Phylogenetic tree of deduced IAG protein sequences in 18 crustacean species. Pf-IAG is indicated with a black arrow. The bar represents the number of amino acid substitutions per site. Bootstrap values (1000 tests) are indicated on the branches.

The deduced structure of the Pf-IAG hormone contain: signal peptide (25 aa), B chain (42 aa), A chain (48 aa) and C peptide (95 aa) [5, 24–27] (Fig 3). A phylogenetic tree of IAG amino acid sequences of 18 species (Fig 4) showed that Pf-IAG bears the closest similarity to the IAG sequence of other crayfish species. More specifically, Pf-IAG shares 100% identity with the IAG sequence of *P*. *clarkii* (Pc-IAG) (accession number KT343750.1) in the A and B amino acid chains, which are the main components of the active IAG protein [5].

### One-shot genomic library of the marbled crayfish

The assembly of the filtered Illumina MiSeq reads (a total of 20,878,594 clean reads with an average length of 142.8 bp) yielded a marbled crayfish one-shot genomic library consisting of 1,133,249 contigs with an average length of 194 bp (min = 17 bp, max = 9,219 bp, SD = 90.2) and an average coverage of 7.5 (min = 1, max = 15,942, SD = 44.7). The genome size of decapod crustacean species in the *Cambarus* genus was reported to be ~5 × 10^9^ bp [28]. Our one-shot genomic library covered 4% of this size. However, for the purpose of mining the *IAG* gene, this preliminary library was sufficient to be used as a reference genome as two contig fragments were found while aligning the *Pf-IAG* mRNA with the aim to target a starting point for genome walking.

### Full genomic sequence of the *IAG* gene in *P*. *fallax* and its corresponding sequence in the marbled crayfish

**Fig 5:**
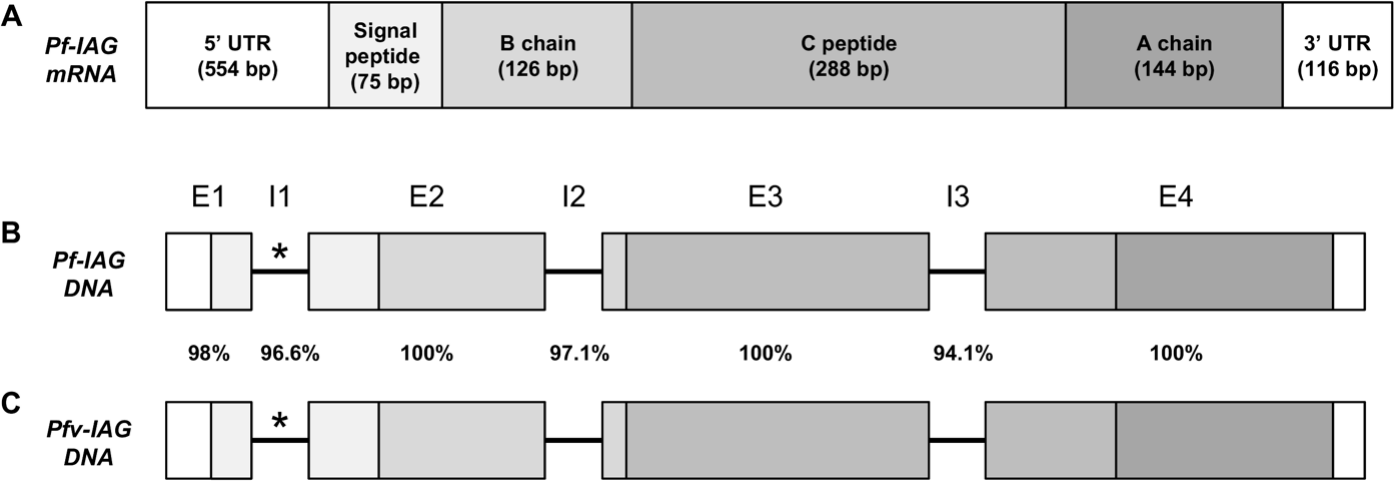
**Structure of *Pf-IAG* mRNA (A) and the genomic structures of *Pf-IAG* (B) and *Pfv-IAG* (C).** The different components of *Pf-IAG* mRNA are colored in different shades of grey, and the length of each component is indicated on the figure. Exons and introns of *Pf-IAG* DNA and their corresponding sequences in the marbled crayfish genome (*Pfv-IAG*) are indicated by the letters 'E' and 'I', respectively. The intron that was not fully sequenced (intron 1) is indicated with an asterisk. The identity (in percentage) of the exons and introns between the two genomic sequences is indicated.

Sequencing the full *Pf-IAG* (accession number MF405196) by genome walking revealed that the gene consists of four exons separated by three introns (Fig 5): exon 1 186 bp), intron 1 (>1747 bp), exon 2 (167 bp), intron 2 (102 bp), exon 3 (212 bp), intron 3 (949 bp) and exon 4 (261 bp). The nucleotide BLAST alignment of *Pf-IAG* mRNA to the marbled crayfish genomic library revealed two fully identical fragments (96 bp of the 5' UTR and 107 bp which is part of both C peptide and A chain). Genome walking upstream and downstream of those fragments revealed a sequence corresponding to that of *Pf-IAG* in the marbled crayfish genome (*Pfv-IAG*; MF405197). This sequence consists of exon 1 (184 bp), intron 1 (>1794 bp), exon 2 (167 bp), intron 2 (102 bp), exon 3 (212 bp), intron 3 (1191 bp) and exon 4 (261 bp). Comparison of the genomic *IAG* sequence in the two species revealed that the open reading frame (ORF) of the marbled crayfish (MF405195) is fully identical to the corresponding sequence in *P*. *fallax*, while the identity of intron 1, intron 2 and intron 3 between the two species is 97%, 97% and 94%, respectively (Fig 5). In both species, intron 1 was not fully sequenced due to a highly repetitive section that was impossible to sequence by the methods used in the present study.

### Spatial expression of *Pf-IAG* in *P*. *fallax* and the marbled crayfish

**Fig 6:**
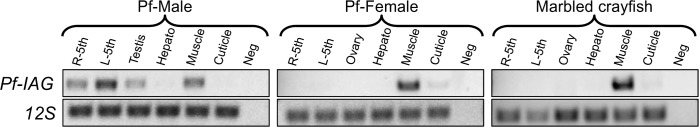
Spatial expression of *Pf-IAG* mRNA in *P*. *fallax* and in the marbled crayfish. RNA was extracted from a *P*. *fallax* male, *P*. *fallax* female and a marbled crayfish from the following tissues: right and left 5th pereiopods (R/L-5^th^), gonad, hepatopancreas, abdominal muscle and cuticle. The negative control (Neg) is shown, and 12S rRNA served as the positive control.

Analysis of the PCR products on a 2% agarose gel (Fig 6) indicated that the masculine gene *Pf-IAG* is expressed, as expected, at the base of the 5th pereiopod (the typical location of the AG) in *P*. *fallax* male. Surprisingly, it was also expressed in the testis of the *P*. *fallax* male as well as in the abdominal muscle of both *P*. *fallax* male and female and even in the abdominal muscle of the parthenogenetic marbled crayfish. Sequencing the observed PCR products confirmed their identity as *IAG* gene. The same pattern of expression was observed in the rest of *P*. *fallax* males, as well as the rest of marbled crayfish animals (S1 Fig).

## Discussion

In the present study, we confirmed that the AG of the crayfish *P*. *fallax* is indeed present in its typical location, the base of the male 5th pereiopod, proximal to the sperm duct [6, 29], and we sequenced the gene encoding for IAG in *P*. *fallax* (*Pf-IAG*). The mRNA sequence of *Pf-IAG*, like the mRNA sequences of the *IAG* genes of other decapod species, was found to contain the well-known components of insulin-like peptides, including a signal peptide, B chain, C peptide and A chain [5, 24, 30, 31]. The phylogenetic tree that we constructed of deduced IAG protein sequences in 18 crustacean species showed that the IAG hormone is highly conserved among crayfish species; for example, the finding that Pf-IAG and Pc-IAG have identical A and B chains is in keeping with the phylogenetic relationship between *P*. *fallax* and *P*. *clarkii*, both species belonging to the North American Cambaridae family [16].

The results of our study offer clear evidence both for the presence of the masculine *IAG* gene in the genome of the all-female marbled crayfish and also for its expression in that parthenogenetic crayfish. The genomic structure of *Pf-IAG*, which contains 4 exons divided by 3 introns, is similar to the full *IAG* gene that has been sequenced in different crustacean species [13]. However, the exact locations and lengths of the introns are different, which indicates that the introns might contain different regulating elements of the IAG gene in the various crustacean species. Moreover, it was impossible to fully sequence intron 1 in both *P*. *fallax* and in the marbled crayfish, as this intron contained highly repetitive sequences. These sequences complicate primer design for genome walking and impose insurmountable difficulties for the Sanger sequencing method used in the present study.

The finding of high similarity (more than 94%) between the introns of *P*. *fallax* and the marbled crayfish, although the ORFs of the *IAG* genes are fully identical, enables us to propose several explanations regarding the evolutionary relationship between the two species. One possible explanation is that the marbled crayfish is a relatively new species that has recently diverged from *P*. *fallax* [14, 17], and, therefore, despite the lack of evolutionary pressure, this gene is conserved and has not undergone significant alterations. Another possible explanation is that the marbled crayfish is actually a virginal form of *P*. *fallax* whose reproductive strategy disregards the *IAG* gene. This assumption is supported by the finding in a different study [32] that *P*. *fallax* males recognize the marbled crayfish female as a potential reproductive partner, but is challenged by the fact that despite the sexual activity of the marbled crayfish, their progeny constitutes an all-female clone [32]. The second explanation is also brought into question by studies suggesting that the marbled crayfish is an independent species, because *P*. *fallax* is a diploid species whereas the marbled crayfish is a triploid organism (with parthenogenesis being suggested to be associated with polyploidy) [18, 32].

Our group previously suggested that the *IAG* gene is present in both male and female genomes, but is exclusively expressed in the male AG [5]. However, on the basis of studies in two species of crab (*Callinectes sapidus* and *Scylla paramamosain*) showing that the *IAG* gene is also expressed in females, it has recently been postulated that IAG is also involved in the processes of growth and reproduction in female crustaceans [12, 13]. Although the above two studies did not detect the peptide or present clear proof of function, the premise that was put forward is supported by the findings in the current study of IAG expression in the muscle tissues of both *P*. *fallax* males and females and of a marbled crayfish animal. In addition, the differences in intron sequences and size (especially in the size of intron 3) between the marbled crayfish and *P*. *fallax* could suggest that different introns regulate the expression patterns of IAG in different roles–in growth processes vs in reproductive processes–and hence support the premise that IAG also serves as a growth factor (which is probably necessary for both parthenogenetic and gonochoristic animals). Finally, the detection of IAG expression in the gonads of *P*. *fallax* males suggests that the IAG might also play a role in testicular developmental processes.

In this study, we report for the first time, the presence of a presumed masculinization-inducing gene–the *IAG*–in a parthenogenetic decapod species. In addition, we found that the ORF of the marbled crayfish was fully identical to the *Pf-IAG* gene and that the gene was expressed in the muscle tissue of the marbled crayfish. These findings make a significant contribution towards characterizing this supposedly androgenic factor not only as an exclusively masculine hormone but also as a functional player in female developmental processes.

## Supporting information

S1 Table List of known 18 IAG sequences in different crustacean species. The sequences from the list were used for phylogenetic analysis of the IAG. GenBank accession numbers are indicated.
(DOCX)

S1 Fig Spatial expression of *Pf-IAG* mRNA in *P*. *fallax* and in the marbled crayfish. RNA was extracted from (A) two more *P*. *fallax* males and (B) five more marbled crayfish from the following tissues: right and left 5th pereiopods (R/L-5^th^), gonad, hepatopancreas, abdominal muscle and cuticle. The negative control (Neg) is shown, and 12S rRNA served as the positive control.
(TIF)
