# Supplementary material for: The gene encoding the insulin-like androgenic gland hormone in an all-female parthenogenetic crayfish
Source: PLoS One. 2017 Dec 20;12(12):e0189982. doi: 10.1371/journal.pone.0189982 (PMC5738133; doi:10.1371/journal.pone.0189982)
Supplement: S1 Table — The sequences from the list were used for phylogenetic analysis of the IAG. GenBank accession numbers are indicated. (DOCX) [file pone.0189982.s001.docx]

Table S1

| **Species** | **Accession number** | **Gene name** |
| --- | --- | --- |
| *Macrobrachium rosenbergii* | FJ409645.1 | *Macrobrachium rosenbergii* insulin-like androgenic gland specific factor (IAG) mRNA, complete cds |
| *Macrobrachium vollenhovenii* | KJ524578.1 | *Macrobrachium vollenhovenii* insulin-like androgenic gland hormone mRNA, complete cds |
| *Macrobrachium nipponense* | KC460325.1 | *Macrobrachium nipponense* insulin-like androgenic gland factor (IAG) mRNA, complete cds |
| *Macrobrachium lar* | AB579012.1 | *Macrobrachium lar* IAG mRNA for insulin-like androgenic gland factor, complete cds |
| *Fenneropenaeus chinensis* | JQ388277.1 | *Fenneropenaeus chinensis* insulin-like androgenic gland hormone isoform 2 (IAG) mRNA, complete cds, alternatively spliced |
| *Sagmariasus verreauxi* | KF220491.1 | *Sagmariasus verreauxi* insulin-like androgenic gland specific factor mRNA, complete cds |
| *Penaeus monodon* | GU208677.1 | *Sagmariasus verreauxi* insulin-like androgenic gland specific factor mRNA, complete cds |
| *Marsupenaeus japonicus* | AB598415.1 | *Marsupenaeus japonicus* IAG mRNA for insulin-like androgenic gland factor, complete cds |
| *Litopenaeus vannamei* | KM066114.1 | *Litopenaeus vannamei* insulin-like androgenic gland hormone precursor, mRNA, partial cds |
| *Callinectes sapidus* | HM594945.1 | *Callinectes sapidus* insulin-like androgenic gland factor (IAGF) mRNA, complete cds |
| *Scylla paramamosain* | JQ681748.1 | *Scylla paramamosain* insulin-like androgenic gland factor (IAG) mRNA, complete cds |
| *Procambarus clarkii* | KT343750.1 | *Procambarus clarkii* insulin-like androgenic gland hormone mRNA, complete cds |
| *Palaemon paucidens* | AB588013.1 | *Palaemon paucidens* IAG mRNA for insulin-like androgenic gland factor, complete cds |
| *Palaemon pacificus* | AB588014.1 | *Palaemon pacificus* IAG mRNA for insulin-like androgenic gland factor, complete cds |
| *Jasus edwardsii* | KF908794.1 | *Jasus edwardsii* insulin-like androgenic gland hormone (IAG) mRNA, complete cds |
| *Cherax quadricarinatus* | DQ851163.1 | *Cherax quadricarinatus* insulin-like androgenic gland factor mRNA, complete cds |
| *Cherax destructor* | EU718788.1 | *Cherax destructor* insulin-like androgenic gland factor mRNA, complete cds |
| *Procambarus fallax* | KX619618.1 | *Procambarus fallax* insulin-like androgenic gland hormone mRNA, complete cds |
