# Supplementary figures and images for: The gene encoding the insulin-like androgenic gland hormone in an all-female parthenogenetic crayfish
Source: PLoS One. 2017 Dec 20;12(12):e0189982. doi: 10.1371/journal.pone.0189982 (PMC5738133; doi:10.1371/journal.pone.0189982)

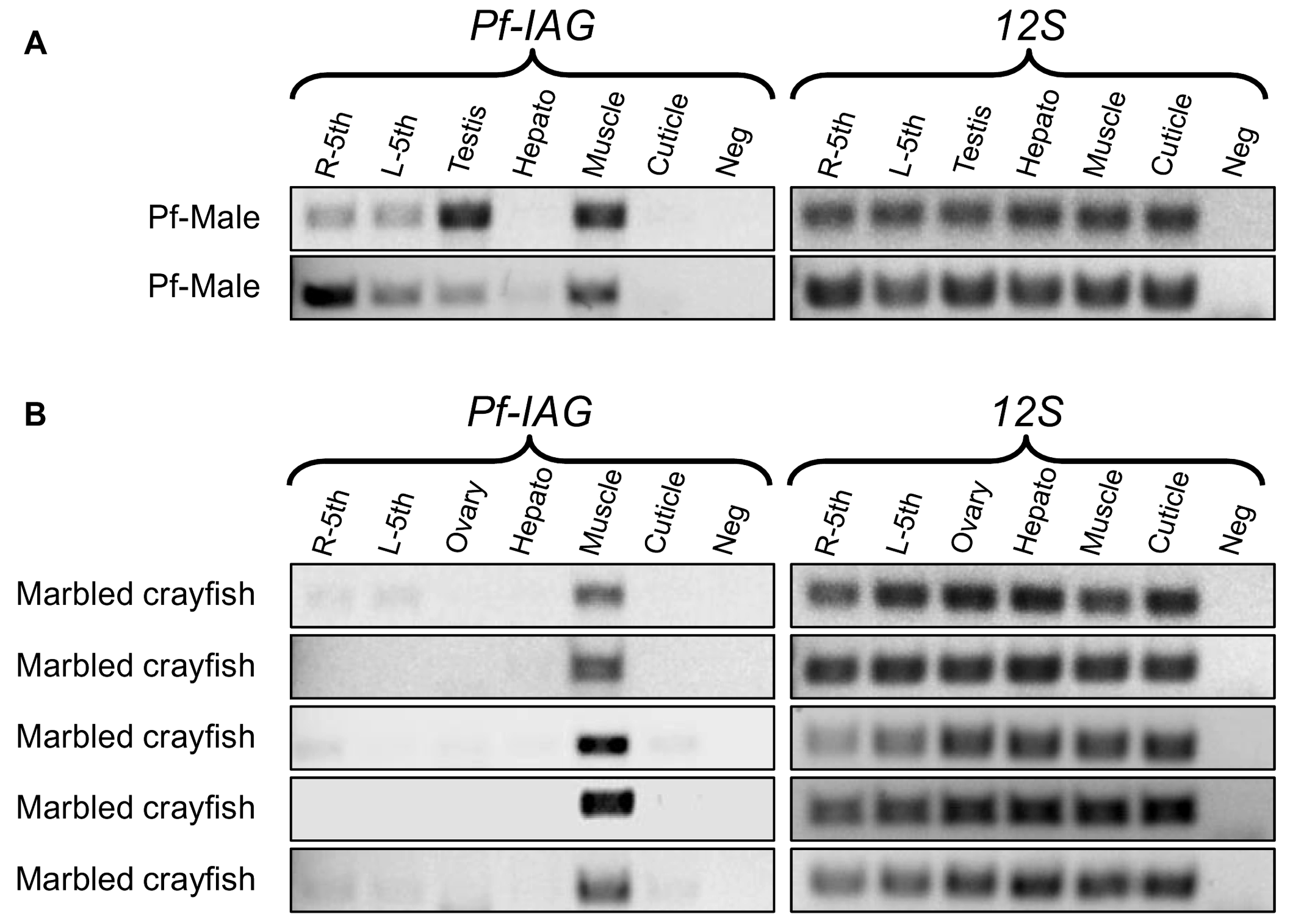

Supplement: S1 Fig — RNA was extracted from (A) two more P. fallax males and (B) five more marbled crayfish from the following tissues: right and left 5th pereiopods (R/L-5th), gonad, hepatopancreas, abdominal muscle and cuticle. The negative control (Neg) is shown, and 12S rRNA served as the positive control. (TIF) [file pone.0189982.s002.tif]
